# Evaluation of long-term stability of monolithic 3D-printed robotic manipulator structures for minimally invasive surgery

**DOI:** 10.1007/s11548-020-02244-6

**Published:** 2020-08-13

**Authors:** Yannick S. Krieger, Daniel Ostler, Korbinian Rzepka, Alexander Meining, Hubertus Feussner, Dirk Wilhelm, Tim C. Lueth

**Affiliations:** 1 Institute of Micro Technology and Medical Device Technology, Technical University of Munich, Munich, Germany; 2 MITI Research Group (Minimally Invasive Interdisciplinary Therapeutical Intervention), Department of General and Visceral Surgery, Technical University of Munich, Munich, Germany; 3 Medical Clinic and Polyclinic II, University Hospital Würzburg, Würzburg, Germany

**Keywords:** Patient-specific, Minimally invasive surgery, Surgical robotics, 3D printing

## Abstract

**Purpose:**

In the era of patient-centered medicine, clinical procedures, tools and instruments should be individually adapted to the patient. In this context, the presented 3D-printed Single-Port Overtube Manipulator System follows the aims to provide patient- and task-specific disposable manipulators for minimally invasive surgery. In a first experiment, the robustness of the monolithic flexure hinge structures in use as robotic manipulators will be investigated.

**Methods:**

Customizable monolithic manipulator structures designed by means of an automated design process and manufactured with selective laser sintering were investigated with regard to long-term stability in an endurance test. Therefore, a bare manipulator arm, an arm equipped with a standard instrument and finally loaded with an additional load of 0.5 N were evaluated by continuously following a trajectory within the workspace of the manipulator arms over a period of 90 min.

**Results:**

The unloaded manipulator as well as the manipulator arm equipped with a standard instrument showed a sufficient reproducibility (deviation of 1.5 mm and 2.5 mm, respectively, on average) with regard to an application as telemanipulated master–slave surgical robotic system. The 3D-printed manipulators showed no damage and maintained integrity after the experiment.

**Conclusion:**

It has been shown that 3D-printed manipulators in principle are suitable for use as disposable surgical manipulator systems and offer a long-term stability over at least 90 min. The developed manipulator design shows great potential for the production of patient-, task- and user-specific robot systems. However, the manipulator geometries as well as the control strategies still show room for improvements.

## Introduction

Minimally invasive surgery (MIS) has gained an importance over the last decade as it offers significant benefits to the patient, such as less patient trauma and a shorter recovery time. The increased application of MIS has led to the development of specific tools to improve the limited vision and access to the surgical site. Furthermore, the principle of MIS has further been improved toward less invasive procedures, e.g., single incision surgery or NOTES [1]. Robotic assistance systems, e.g., the da Vinci single- and multi-port systems (Intuitive Surgical, Sunnyvale, USA), follow principles of MIS. As a flexible robotic system, the FLEX Robotic system (Medrobotics, Raynham, USA) is available as well as several other systems are currently being developed in research as outlined by [2, 3]. The available systems as well as those in development that are usually designed as complex and fully defined universal robots, which can only minimally be adapted to varying interventions and anatomies. As an alternative solution, the Single-Port Manipulator System (SPOT) is presented as an innovative approach for customizable manipulators for varying MIS applications, which is developed as part of an interdisciplinary research project (DFG FOR 1321).

The SPOT Manipulator System is designed as an overtube for standard flexible or rigid endoscopes with adaptable manipulator arms at its distal end. Standard endoscopic instruments can be inserted in the working channels and then be manipulated by the manipulator arms. Beside the general production of such a system, our main goal is the development of a manipulator design, which can be fully adapted to the patient and the surgeon’s needs as well as the intended application [4]. To achieve this, an automated design process for the manipulator structures based on a monolithic flexure hinge design was developed. The monolithic manipulator structures consist of flexure hinges, which define the manipulator’s kinematics and compliance, and of rigid structural elements, which define the shape and additional functional characteristics. The monolithic structure does not have to be assembled. Only the Bowden wires must be mounted into the 3D-printed structure. Furthermore, flexure hinges show advantages over classical rotation joints such as good sterilizability and the capacity to be utilized in small-scale applications. Depending on the required forces and the application, the individual flexure hinges can be adapted  [5] as well as the entire shape of the manipulator, e.g., the entire shaft design. The manufacturing is done using selective laser sintering (SLS) with polyamide 12 (PA2200, EOS, Kailling, Germany), which is biocompatible according to EN ISO 10993-1. Therefore, SPOT manipulators are soft robots that are extrinsically soft, i.e., their compliance is achieved by their structural design rather than by manufacturing them from soft materials. In order to optimally adapt the system to the requirements of the application and the needs of the operating surgeon, different control concepts for the soft robotic manipulator structures have been investigated such as purely mechanical direct drive principles for an endoscopic SPOT manipulator as published in [4]. A new compact electrical control approach based on smart servos and a double cable system for Bowden cable actuation of the monolithic manipulator structures will be presented in the following. In combination with the control concept, the robustness of the manipulator structures under the increased loads caused by the electrical actuation by means of double pull is to be investigated.

## Materials and methods

**Fig. 1:**
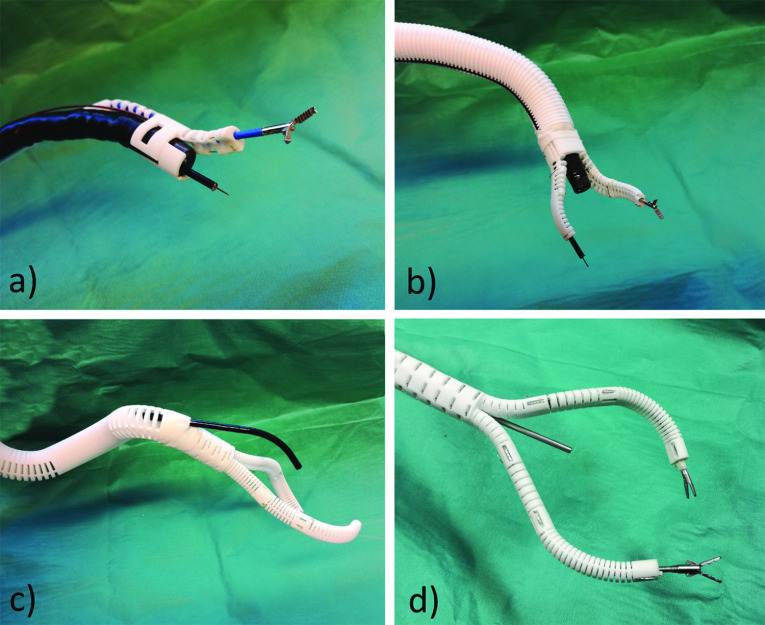
Examples of various SPOT manipulator structures: **a** slim single-arm manipulator for gastroenterology mounted on a flexible standard endoscope; **b** two arm manipulator for gastroenterology with shaft structure that can be made stiff; **c** laparoscopic manipulator with a semi-shaft that can assume an predefined pose; **d** laparoscopic manipulator with straight rigid shaft design and a rigid optic

Figure 1 shows SPOT manipulators which were individually designed to suit different applications. In addition to the number of manipulator arms and their working range, the shaft design (flexible, stiffenable, predefined shape or rigid (see Fig. 1) or additional features such as an optional camera can be customized.

**Fig. 2:**
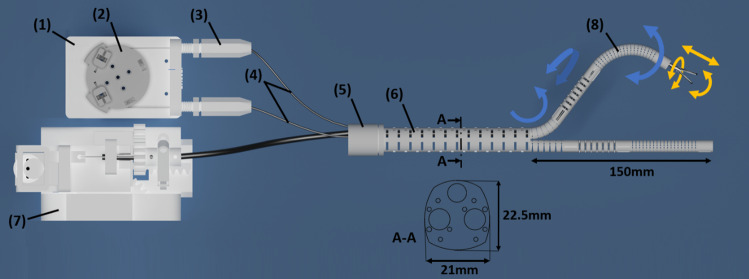
Schematic representation of a system design with rigid shaft. As an example, the actuation module of one degree of freedom of the left manipulator arm and the control unit for the left instrument is shown schematically. Each degree of freedom is controlled by a motor module (1) with a circular transmission element (2) via two wires. The Bowden cables (4) are pretensioned by the adjustment mechanism (3). The sealing element (5) is attached to the proximal end of the rigid shaft (6). At the distal end of the shaft are the two manipulator arms (8) with 3 DoF each (blue). Additionally, the instrument guided in the working channel is manipulated with 3 DoF (orange) when actuated by the instrument actuation module (7)

**Fig. 3:**
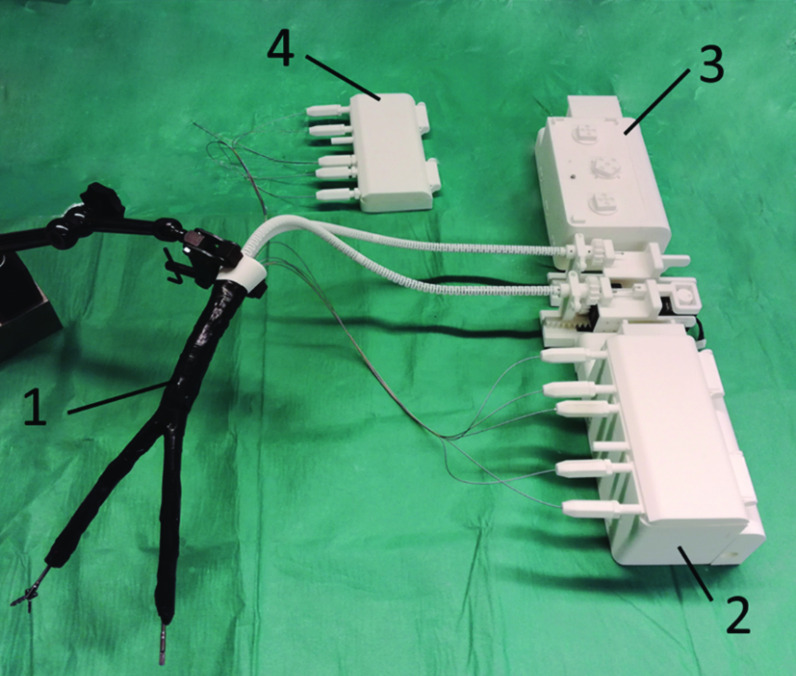
System components: (1) SPOT manipulator system with rigid shaft and black silicone cover; (2) electrical control unit for the left manipulator arm; (3) motor box of the right manipulator arm with quick coupling adapters; (4) coupling adapter of right arm

With regard to the automated design approach, a suitable concept for the integration of electrical control units for the individualized manipulator structures was implemented. A control unit using integrated robot servos (FeeTech, Shenzhen, China) was designed that can be adapted to the manipulator type and the required forces. The motors are housed in a single-motor box, which serves as the system base (see Fig. 3 (3)) with integrated quick coupling adapters. The counterpart of the quick couplings is the coupling elements of each manipulator arm (see Fig. 3 (4)), which each contain a transmission element (see Fig. 2 (2)) for the actuation of each DoF of the arm. Due to this quick coupling design, it is possible for the system to be used in a sterile environment when the motor box is covered with a sterile foil and then attach to the sterilized manipulator via the coupling adapters. In addition to the already published mechanical control [4, 5], the new model thus offers electrical control via adaptable double-pull mechanisms.

Each DoF, represented by a flexure hinge chain, is actuated by two traction cables which are deflected in opposite directions. This actuation concept was introduced especially for large manipulator types requiring higher forces. The much higher tensile loading in the Bowden cables compared to used push rod design in [4] is compensated through the integration Bowden cables which are optimized for compression (tension spring strands with spring strength 0.35 mm made of 1.4301). The system is sealed by a 50-µm-thick TPU foil (Fait Plast, Cellatica, Italy) in combination with an individualized silicone sealing plate in the sealing element (see Fig. 2 (5)). The laparoscopic SPOT manipulator with rigid shaft (see Figs. 1d and 3) described in this manuscript measures 21 mm × 22.5 mm and thus is comparable to other Single-Port systems (e.g., Da Vinci SP). Figure 2 schematically shows the actuation concept as well as the design of the manipulator.

Each arm can be controlled using a 3D mouse, which represents similar DoF compared to the purely mechanical control units described in [4]. Due to the kinematic design of the manipulator arms, which is adapted to the anatomy of the human arm (2 DoF shoulder, 1 DoF elbow), the telemanipulated control under endoscopic view in joint space is intuitive.

## Experiment

Using such a customizable 3D-printed robotic system for a surgical procedure has many potential benefits. However, its integrity over longer lasting operations has not been proven so far. Specifically, the stability of the 3D-printed flexure hinge structures requires evaluation. It is to be examined whether the individual joints and the entire system are maintained in their kinematics and steerability during an operation. To assess these issues, in this study we tested the repeatability of motion and long-time stability for the described compliant monolithic manipulators. Using an optical tracking system (Vicra Polaris, NDI, Ontario, Canada), the consistency of movements of a bare manipulator arm was measured first. Then a flexible forceps instrument (SPIDER, TransEnterix, Morrisville, USA) was loaded into the working channel and the tip of the instrument was tracked. Finally, an additional load of 0.5 N was then applied to the instrument tip. The accuracy and consistency of movements was evaluated with the tracking device over a duration of 90 min. Movements followed a pre-programmed trajectory with a rectangular shape which were computer-controlled via the motor activation.

## Results

**Fig. 4:**
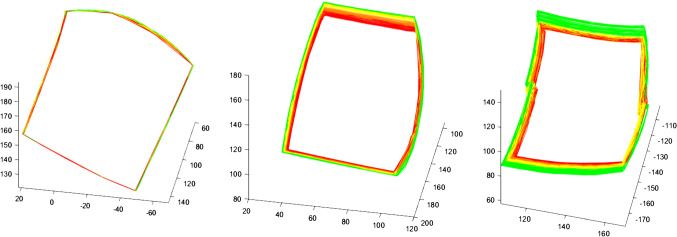
Consistency of movements over 90 min following a trajectory in work space. The colors from green to red show the trend over time: (left) Movements of the tip of the bare manipulator arm; (middle) movements of the loaded manipulator arm; (right) movements of the instrument’s tip with an additional load of 0.5 N

Resulting movements for the three setups are displayed in Fig. 4. For the unloaded manipulator, the measured values deviated by 1.5 mm on average, whereas the loaded system showed deviations of 2.5 mm on average. Additionally, loading the system with 0.5 N resulted in a deviation of 4.5 mm on average. Since the position of the optical trackers had to be changed during the different measurements, the trajectories are positioned differently in space. As can be seen, the precision and consistency of movements are obviously influenced by the additional load, especially at the edges of the working area. The 3D-printed manipulators showed no damage after the experiment after inspection.

## Discussion

In this study, the repeatability of the movements of a soft robotic manipulator was investigated to assess the suitability of 3D-printed monolithic flexural hinge structures for use in surgical robots. Repeatability is also a decisive criterion for use in master–slave systems, where the human is kept in the control loop at all times [6]. We interpret our results as very promising as the system followed the given trajectory with acceptable consistency over a period of 90 min. Measurements of the intrinsic unloaded accuracy of the da Vinci S system shown in [7] (1.05 ± 0.24 mm) can serve as a rough reference for the comparison to our measured values, since there are no concrete comparative values for reproducibility of movements for existing robots in visceral surgery [6]. These values are of comparable order of magnitude. Admittedly, we observed a remarkable deviation over time when loading the system; however, the aberration appeared within the first third of the course and then remained nearly stable. Thus, we believe the deviation is mainly caused by lengthening and setting of Bowden wires, which can easily be compensated for by an adaption of the electronic control. This assumption is further supported by visual inspection of the systems after the experiments, which showed no damage. Therefore, it suggests that our results, especially as investigated in a soft robotic system, are in an acceptable range and compensable by applying an adaptive computer control system. The observed deviation, besides the setting and lengthening of Bowden wires, might also be attributed to the electrical system, i.e., the servo motors. The motors heated up remarkably during use and reached their limits, especially when loaded with an additional weight, although the holding force of the manipulator is 2.5 N. The heating of the motors resulted in a reduced torque, which in turn impacted on the movement trajectory. Although we did not measure the temperature of the servos during the scenario and thus cannot prove a correlation of heating and altered trajectory, we assume this to be a potential supplementary cause of deviation. Although it is questionable whether the continuous movement of tissue as applied in this scenario is realistic, we will use stronger servo motors in the future.

## Conclusion

This study for the first time showed the applicability of monolithic 3D-printed surgical robots for in vitro long-term use over 90 min. With the positive assessment of the presented monolithic structures, this offers fundamental new design possibilities for disposable surgical manipulators, allowing patient-specific (e.g., length, workspace, max. forces) and task-specific (e.g., access route, flexible vs. rigid) configurations. The already acceptable stiffness can even be improved by integration of rigid or locking elements. The customizable and (semi-)flexible design using cost-effective 3D printing technology could provide surgeons with highly effective and individualized surgical manipulators which will allow for the development of completely new approaches and further reduction in the interventional trauma. Further studies are deemed warranted.
